# Psychological Dimensions of Professional Burnout in Special Education: A Cross-Sectional Behavioral Data Analysis of Emotional Exhaustion, Personal Achievement, and Depersonalization

**DOI:** 10.3390/ijerph22091420

**Published:** 2025-09-11

**Authors:** Paraskevi-Spyridoula Alexaki, Hera Antonopoulou, Evgenia Gkintoni, Nikos Adamopoulos, Constantinos Halkiopoulos

**Affiliations:** 1 Department of Management Science and Technology, University of Patras, 265 04 Patras, Greece; up1089427@upatras.gr (P.-S.A.); hera@upatras.gr (H.A.); nadamop@upatras.gr (N.A.); 2 University General Hospital of Patras, 265 04 Patras, Greece; evigintoni@upatras.gr

**Keywords:** professional burnout, special education teachers, behavioral data analysis, emotional exhaustion, personal achievement, depersonalization, COVID-19 impact, occupational health

## Abstract

Background and Objectives: Professional burnout threatens special education teachers’ well-being and educational service quality through three psychological dimensions: emotional exhaustion, depersonalization, and personal achievement. Limited studies have employed behavioral data analysis to examine burnout patterns in special education and their relationships with demographic factors and contemporary stressors. This study aimed to (1) identify burnout levels among Greek special education teachers, (2) determine demographic risk factors, and (3) examine relationships between burnout dimensions and COVID-19 psychological impact. Materials and Methods: A cross-sectional study surveyed 114 special education teachers from Achaia and Aitoloakarnania prefectures, Greece (response rate: 87.7%), using the Maslach Burnout Inventory–Educators Survey (MBI-ES) and demographic questionnaires. Behavioral data analysis integrates traditional statistics with advanced techniques, including cluster analysis and classification modeling. Results: Four distinct burnout profiles emerged: Low Burnout (36.8%), Moderate Emotional Exhaustion (30.7%), High Risk (21.9%), and Depersonalization-Dominant (10.5%). Overall burnout prevalence was low, with 73.7% showing minimal depersonalization and 67.5% maintaining high personal achievement. Employment status emerged as the strongest predictor of burnout risk. Emotional exhaustion was the primary predictor of COVID-19 psychological impact (*r* = 0.547, *p* < 0.001), explaining 29.9% of pandemic-related distress variance. Male substitute teachers demonstrated the highest vulnerability to depersonalization, while experienced female permanent teachers showed resilience patterns. Conclusions: Behavioral data analysis revealed distinct burnout patterns enabling personalized interventions. Emotional exhaustion serves as both a key vulnerability factor and primary intervention target. These findings support targeted approaches to occupational health with implications for educational policy. Limitations include cross-sectional design and regional sampling. Future longitudinal studies should validate these patterns across diverse educational contexts.

## 1. Introduction

Professional burnout represents a critical occupational hazard that has garnered increasing attention within educational research, particularly in the specialized field of special education. Characterized by three distinct psychological dimensions—emotional exhaustion, depersonalization, and reduced personal achievement—burnout syndrome poses significant threats to educator well-being, instructional quality, and student outcomes in special education settings [1,2,3,4].

The unique challenges inherent in special education environments create a constellation of risk factors that distinguish these settings from mainstream educational contexts [5,6,7]. Special education teachers navigate complex demands, including intensive behavioral management, diverse student needs across multiple disability categories, administrative bureaucracy, and heightened emotional labor requirements. These multifaceted stressors contribute to elevated burnout rates among special education professionals, with research consistently documenting higher incidence rates compared to general education counterparts [8,9,10,11,12].

### 1.1. The Three Dimensions of Burnout

Contemporary burnout research has established three core psychological dimensions that collectively define the syndrome. Emotional exhaustion manifests as depletion of emotional resources and chronic fatigue resulting from excessive psychological demands. Depersonalization involves the development of cynical attitudes and emotional detachment from students and colleagues. Personal achievement reflects educators’ self-evaluation of professional competence and effectiveness in their educational roles [13,14,15].

Understanding these dimensions and their relationships with demographic factors offers significant opportunities to identify patterns and predictive indicators of burnout development. Cross-sectional analyses enable researchers to capture the multidimensional nature of burnout while examining demographic, organizational, and contextual variables that moderate these relationships. Research has identified five main educational demands that influence burnout levels: esteem, sociability, security, self-realization, and autonomy [16,17,18].

### 1.2. Risk Factors in Special Education Settings

The special education environment presents unique challenges that increase the risk of burnout. Research has consistently identified several key contributing factors.

Student-related factors include working with students exhibiting emotional and behavioral disturbances, which increases stress exposure. Classroom composition variables—including student age ranges (particularly adolescent populations aged 13–19), disability categories, and class sizes—significantly influence burnout levels [19,20,21,22].

Organizational factors encompass administrative challenges such as inadequate support, role ambiguity, excessive bureaucracy, and resource limitations that exacerbate psychological strain. The gap between pre-service expectations and actual working conditions often exacerbates the development of burnout among novice special educators [23,24,25,26].

Demographic factors reveal important patterns. Gender differences have emerged as significant predictors, with male educators demonstrating higher rates of depersonalization and emotional exhaustion. Teaching experience shows complex relationships with burnout dimensions, with both minimal and extensive seniority presenting unique challenges [27,28].

### 1.3. International Perspectives and Research Gap

While burnout research in special education has evolved since the 1990s, significant gaps remain in our understanding of the psychological mechanisms underlying these three dimensions. International research efforts have documented varying patterns across different contexts, yet these studies often lack integration and critical synthesis [29,30]. European findings reveal converging patterns. Studies from Ireland have shown relationships between burnout and student problem behavior, with classroom-level factors such as student indiscipline and disruptive behavior repeatedly identified as significant predictors of burnout [31]. Israeli research examining special education teachers identified complex relationships between seniority and burnout dimensions, with both novice and veteran teachers facing unique stress challenges [32]. UK investigations found that administrative bureaucracy, workload, and time constraints were positively associated with burnout, with 37% of senior educational leaders showing the highest signs of burnout compared to 27% of classroom teachers [33]. Turkish research confirmed burnout as a common phenomenon in special education, with teachers experiencing very high levels of burnout, and studies showing that male teachers exhibited less emotional exhaustion but higher depersonalization than their female counterparts [34,35]. Critical synthesis of these international studies reveals two key patterns: (1) organizational factors (bureaucracy, workload) consistently predict burnout across cultures, and (2) gender differences in burnout manifestation appear universal, though their expression varies by cultural context [36,37,38].

### 1.4. Contemporary Challenges in Special Education

Recent global health crises, particularly the COVID-19 pandemic, have introduced unprecedented challenges to educational systems worldwide. Special education teachers faced unique difficulties in maintaining specialized services during remote learning periods, potentially exacerbating existing burnout vulnerabilities. Understanding how pre-existing burnout levels influence teachers’ responses to such contemporary stressors represents a critical gap in the literature. This relationship has important implications for supporting educator resilience during future crises. Limited cross-sectional studies have examined the interplay between demographic variables and burnout dimensions in special education contexts. While behavioral data mining has shown promise in other occupational health domains, its application to educational burnout—particularly in revealing how neurocognitive factors, digital competencies, and traditional stressors interact—represents a critical gap in the literature. Recent global health crises have introduced additional psychological stressors that may interact with traditional burnout factors in previously unexplored ways, making the need for sophisticated analytical approaches even more pressing [39].

### 1.5. The Greek Context and Study Rationale

Greece presents a particularly relevant context for studying special education teacher burnout due to several unique factors. The Greek educational system has undergone significant reforms in special education over the past decade through EU-funded technical support projects aimed at transforming the educational system toward inclusive education, creating both opportunities and challenges for educators [40,41]. Economic constraints following the financial crisis have impacted resource allocation and job security, with many special education positions filled by substitute rather than permanent teachers [42,43]. Additionally, Greek cultural values emphasizing collective responsibility and emotional investment in education may influence how burnout manifests compared to more individualistic societies [44]. The recent implementation of inclusive education policies, particularly Law 4368/2016, which emphasizes full inclusion of children with special education needs in mainstream classrooms, has increased demands on special education teachers requiring them to support pupils through differentiated activities and practices without corresponding increases in support structures [45,46]. These systemic and cultural particularities make Greece an ideal setting for examining how burnout develops under conditions of institutional change and resource constraints [47,48].

### 1.6. Scope of the Research

This study addresses these critical gaps through a comprehensive cross-sectional analysis examining the psychological dimensions of professional burnout among special education professionals. The research scope encompasses the systematic investigation of the three core burnout dimensions—emotional exhaustion, depersonalization, and personal achievement—within the specialized context of special education environments.

The investigation extends beyond simple prevalence documentation to explore the complex interrelationships between burnout dimensions and demographic variables, including gender, age, teaching experience, educational background, and workplace characteristics. Additionally, the research incorporates contemporary contextual factors, specifically examining how recent global health crises have influenced the psychological burden experienced by special education professionals and how these stressors interact with existing patterns of burnout.

### 1.7. Research Questions

The research questions that are posed to investigate the purpose and the individual objectives are the following:

- [RQ1] What are the levels at which the dimensions of burnout are observed in the sample? This question seeks to establish baseline prevalence rates for emotional exhaustion, depersonalization, and personal achievement among special education professionals, providing foundational descriptive data for the target population.
- [RQ2] What is the degree of correlation between the three dimensions of burnout and demographic factors? This investigation examines the relationships between burnout dimensions and key demographic variables, including gender, age, teaching experience, educational background, and workplace characteristics, to identify at-risk populations and protective factors.
- [RQ3] What is the degree of impact of the three dimensions of burnout on the psychological burden of the individual due to the recent health crisis? This question examines how pre-existing burnout dimensions affect educators’ psychological responses to contemporary health-related stressors, exploring burnout as a vulnerability factor.
- [RQ4] What is the degree of impact of psychological burden due to the health crisis as a function of all three dimensions of burnout? This inquiry investigates the combined predictive power of all three burnout dimensions in explaining variance in psychological burden related to health crises, examining the cumulative and interactive effects of the burnout syndrome on contemporary stress responses.

## 2. Materials and Methods

### 2.1. Research Design and Purpose

This study employed a quantitative cross-sectional design to investigate the presence of burnout among special education teachers in primary education school units within the prefectures of Achaia and Aitoloakarnania, Greece. The research aimed to examine the psychological burden experienced by these educators due to recent health crises and to identify potential correlations between burnout dimensions and demographic factors. Quantitative approaches were selected based on their ability to provide measurable empirical data through predetermined research designs, enabling the standardized and unbiased examination of burnout-related theories [47]. This methodological approach facilitates the interpretation of results by comparing them with previous research and ensures consistency in data collection and analysis procedures.

### 2.2. Participants and Sampling

The target population comprised special education teachers working in primary education settings within the prefectures of Achaia and Aitoloakarnania. A convenience sampling method was employed due to practical constraints, including limited access to complete population registries, time restrictions during the academic year, and the voluntary nature of participation required by ethical guidelines. While convenience sampling may limit generalizability, it was deemed appropriate for this cross-sectional study, as our primary goal was to identify patterns. The final sample of 114 participants achieved good coverage, with a response rate of 87.7% (114 completed questionnaires out of 130 distributed), suggesting minimal non-response bias within the accessible population.

Demographic characteristics revealed that 30 participants (26.3%) were male and 84 (73.7%) were female, reflecting the gender distribution typical of the teaching profession. The predominant age group was 31–35 years, representing 28.1% of the sample. Educational attainment was high, with 49.1% of participants holding at least one postgraduate degree and 89.1% possessing degrees from Higher Education Institutions (HEI) or Technological Educational Institutions (TEI). Regarding marital status, 60.5% of participants were married, providing insight into the life circumstances of the study population.

### 2.3. Data Collection Procedures

Data collection was conducted between 8 January and 2 February 2022, using an anonymous electronic questionnaire distributed via email. The questionnaire was designed and administered through the Google Forms platform, ensuring accessibility and ease of completion. The researcher maintained ethical standards by contacting participants only once, with minimal follow-up limited to technical issues on the first day of distribution when some recipients experienced difficulties accessing the electronic link.

Personal contact was minimal, preserving anonymity while ensuring a high response rate. The questionnaire required approximately ten minutes to complete, minimizing participant burden while maintaining comprehensive data collection. Initial communication included a formal letter to school units or headmasters’ personal emails, clearly stating the researcher’s credentials, study purpose, and providing the unique electronic link for questionnaire access.

### 2.4. Instrumentation

#### 2.4.1. Demographic Questionnaire

Participant characteristics were assessed using a custom demographic questionnaire targeting variables identified in the literature as potential correlates of teacher burnout [48]. The questionnaire collected information on age, gender, additional studies, marital status, school location, number of departments in the school unit, years of service in education, and years of service as a special education teacher.

Additionally, a COVID-19 psychological impact assessment was conducted. Participants were asked to respond to the following item: “To what extent has the COVID-19 pandemic affected your psychological state?” Responses were collected using a continuous percentage scale from 0% (no impact) to 100% (extreme impact), allowing participants to indicate their perceived psychological burden with precision. This variable was treated as continuous in regression analyses and correlation calculations, providing a nuanced measure of pandemic-related stress. The single-item percentage format was chosen for its intuitive interpretation and to minimize assessment burden while capturing subjective psychological impact.

#### 2.4.2. Maslach Burnout Inventory–Educators Survey (MBI-ES)

Burnout assessment utilized the Maslach Burnout Inventory–Educators Survey (MBI-ES) developed by Maslach, Jackson, and Schwab (1996) [49], translated and adapted into Greek by Kokkinos (2002) [50]. The MBI-ES represents the most recent version specifically adapted for educational settings and is the most widely used burnout measurement instrument internationally, employed in approximately 90% of relevant research studies [51]. The Greek version maintains the same internal consistency and factorial structure as the original American version [52], ensuring international comparability of findings.

The instrument comprises 21 self-assessment items distributed across three subscales corresponding to the three burnout dimensions: emotional exhaustion (10 items), personal achievement (6 items), and depersonalization (5 items). Each subscale operates independently without composite scoring. Minor modifications were implemented to target primary special education teachers specifically.

Responses were measured using a seven-point Likert scale (0–6) assessing frequency of occurrence, where 0 = never; 1 = a few times a year or less; 2 = once a month or less; 3 = a few times a month; 4 = once a week; 5 = a few times a week; and 6 = every day. Burnout severity for each dimension corresponds to the mean response score across relevant items.

#### 2.4.3. Scoring and Interpretation

For the primary analyses in this study, we utilized established threshold values for burnout levels based on summed scores: emotional exhaustion (low ≤20, medium 21–40, high ≥41); lack of personal achievement (low ≤12, medium 13–24, high ≥25); and depersonalization (low ≤10, medium 11–20, high ≥21). It is important to note that the personal achievement scale was reverse-coded to measure “lack of personal achievement”, ensuring consistent interpretation where higher scores indicate greater burnout across all three dimensions. This transformation aligns with the theoretical framework where burnout is characterized by high emotional exhaustion, high depersonalization, and low personal achievement.

High burnout is characterized by elevated scores of emotional exhaustion and depersonalization combined with low personal achievement scores (high lack of personal achievement after reversal), while low burnout presents the inverse pattern.

#### 2.4.4. Reliability and Validity

The MBI-ES demonstrates established psychometric properties, with Cronbach’s alpha reliability coefficients reported by the original authors as follows: emotional exhaustion (α = 0.90), personal achievement (α = 0.71), and depersonalization (α = 0.79). Greek validation studies have confirmed instrument reliability with α values of 0.80, 0.90, and 0.68 [50]. In the present study, reliability analysis yielded satisfactory Cronbach’s alpha coefficients, exceeding the 0.70 threshold: emotional exhaustion (α = 0.861), personal achievement (α = 0.865), and depersonalization (α = 0.794). These results confirm the instrument’s reliability for the current sample.

### 2.5. Data Analysis

Statistical analyses were conducted using SPSS 22.0 software with significance set at *p* < 0.05. The analytical approach was designed to address each research question directly:

- For RQ1 (burnout levels): Descriptive statistics included means, standard deviations, minimum and maximum values, and frequency distributions. Numerical and ordinal variables were analyzed using means, standard deviations, and bar charts, while categorical variables were examined through frequency tables, percentage bar charts, and pie charts.
- For RQ2 (demographic correlations): Inferential analyses employed Pearson’s linear correlation coefficient to examine relationships between numerical and ordinal variables. This addressed the relationships between burnout dimensions and demographic factors.
- For RQ3 and RQ4 (COVID-19 impact): Simple linear regression was utilized to assess the impact of burnout dimensions on psychological burden related to the health crisis. The internal consistency of the three burnout subscales was evaluated using Cronbach’s alpha coefficient, with values greater than 0.70 indicating satisfactory reliability.

#### 2.5.1. Additional Statistical Procedures

To provide deeper insights into burnout patterns, additional analyses were conducted using R version 4.1.2 (R Core Team, 2021. R: A Language and Environment for Statistical Computing. R Foundation for Statistical Computing, Vienna, Austria).

- Cluster Analysis: K-means clustering was performed to identify distinct burnout profiles using the three burnout dimensions as input variables. The optimal number of clusters was determined through the elbow method and silhouette analysis.
- Classification Analysis: Decision tree analysis using the CART algorithm identified demographic and workplace factors that predict burnout risk. This approach provided clear decision rules for identifying at-risk teachers.
- Advanced Regression Techniques: To examine potential non-linear relationships between burnout dimensions and COVID-19 psychological impact, multiple modeling approaches were compared.

Before implementing data mining algorithms, comprehensive data preprocessing was conducted to ensure optimal analysis conditions. Missing data handling: Little’s MCAR test indicated that missing values (<2% across all variables) were completely at random (χ^2^ = 23.45, *p* = 0.76). These were imputed using predictive mean matching to preserve distributional properties. Outlier detection: the interquartile range (IQR) method identified 3 extreme outliers in the COVID-19 impact variable, which were Winsorized to the 95th percentile to retain information while reducing influence. Standardization: all continuous variables were z-score standardized (mean = 0, SD = 1) to ensure comparability across different scales.

#### 2.5.2. Missing Data and Assumptions

Little’s MCAR test assessed whether missing data (<2% across all variables) were missing completely at random. Missing values were handled using pairwise deletion for correlation analyses and listwise deletion for regression analyses. All statistical assumptions (normality, linearity, homoscedasticity) were tested before conducting parametric analyses. Where assumptions were violated, appropriate non-parametric alternatives or data transformations were applied.

### 2.6. Ethical Considerations

Ethical standards were maintained throughout the research process, adhering to the principles of survey research ethics. The introductory section of each questionnaire explicitly guaranteed confidentiality, anonymity, and privacy of personal data. Participation was voluntary, and no identifying information was collected. The electronic format enhanced anonymity protection while enabling participants to complete the questionnaire at their convenience. The researcher maintained professional boundaries by limiting contact to essential communications and respecting participants’ privacy throughout the data collection period.

## 3. Results

### 3.1. Participant Characteristics

The final sample consisted of 114 special education teachers from the prefectures of Achaia and Aitoloakarnania. As shown in Table 1, demographic analysis revealed a predominantly female sample (*n* = 84, 73.7%) compared to male participants (*n* = 30, 26.3%). The most represented age group was 31–35 years (*n* = 32, 28.1%), while the smallest group was under 25 years (*n* = 6, 5.3%). Educational attainment was high, with 49.1% (*n* = 56) holding postgraduate degrees and 37.7% (*n* = 43) holding university degrees. The majority of participants were married (60.5%, *n* = 69), with 35.1% (*n* = 40) being unmarried. Geographically, 61.4% (*n* = 70) worked in Achaia prefecture and 38.6% (*n* = 44) in Aitoloakarnania prefecture. Employment status was relatively balanced between permanent teachers (52.6%, *n* = 60) and substitute teachers (47.4%, *n* = 54).

Regarding health and recent life events, 23.7% (*n* = 27) reported being affected by COVID-19 in the past year, while 14.0% (*n* = 16) experienced the loss of a family member or friend. Half of the participants (50.0%, *n* = 57) selected “other” for recent problems, indicating either no significant issues or concerns not listed in the questionnaire. For chronic health conditions, autoimmune diseases were most prevalent (9.6%, *n* = 11), followed by disabilities (7.0%, *n* = 8) and mental health conditions (5.3%, *n* = 6).

### 3.2. Burnout Inventory Analysis

#### 3.2.1. Emotional Exhaustion

Analysis of the emotional exhaustion subscale revealed a mean total score of 23.31 (SD = 16.74, range = 0–60), indicating moderate levels of emotional exhaustion among participants (detailed item analysis of Table S1 is available in the Supplementary Materials). The highest-scoring item was “I feel like I’m working too hard at school” (*M* = 3.40, SD = 1.77), while the lowest was “I feel full of energy” (*M* = 1.74, SD = 1.34). Internal consistency for this subscale was excellent (Cronbach’s α = 0.865). Categorical analysis of emotional exhaustion levels (see Table 2) showed that 45.6% (*n* = 52) of participants experienced low emotional exhaustion, 44.7% (*n* = 51) experienced moderate levels, and 9.6% (*n* = 11) experienced high emotional exhaustion.

#### 3.2.2. Personal Achievement

The personal achievement subscale, reverse-coded to measure lack of personal achievement for consistency with other burnout dimensions, yielded a mean total score of 10.33 (SD = 8.64, range = 0–34). Lower scores on this reversed scale indicate higher personal achievement (a positive outcome) (detailed item analysis of Table S2 is available in the Supplementary Materials). The subscale demonstrated good internal consistency (Cronbach’s α = 0.794).

#### 3.2.3. Depersonalization

The depersonalization subscale produced a mean total score of 6.79 (SD = 7.84, range = 0–29), indicating low levels of depersonalization across the sample (detailed item analysis of Table S3 is available in the Supplementary Materials). Internal consistency was satisfactory (Cronbach’s α = 0.794).

#### 3.2.4. Overall Burnout Profile

The overall MBI-ES total score averaged 40.43 (SD = 33.22, range = 0–117), suggesting generally low burnout levels across the sample.

### 3.3. Statistical Analysis (RQ2: Demographic Relationships)

#### 3.3.1. Comprehensive Correlation Analysis

A comprehensive correlation matrix was computed to examine relationships among all study variables, including demographic factors, burnout dimensions, and COVID-19 psychological impact. Table 3 presents the complete correlation matrix, revealing several significant associations.

The correlation analysis revealed several noteworthy patterns. Age and years of experience showed a very strong positive correlation (*r* = 0.847, *p* < 0.01), as expected. Gender demonstrated significant associations with depersonalization (*r* = 0.287, *p* < 0.01), indicating that male teachers reported higher levels of depersonalization. Employment status correlated negatively with depersonalization (*r* = −0.267, *p* < 0.01), suggesting that permanent teachers experience less depersonalization than substitute teachers. Among burnout dimensions, emotional exhaustion and depersonalization showed a moderate positive correlation (*r* = 0.456, *p* < 0.01), while personal achievement correlated negatively with both emotional exhaustion (*r* = −0.234, *p* < 0.05) and depersonalization (*r* = −0.189, *p* < 0.05), indicating the expected inverse relationships between personal accomplishment and other burnout dimensions.

#### 3.3.2. Demographic Group Comparisons

Independent samples *t*-tests were conducted to examine gender differences in burnout dimensions, as shown in Table 4. Significant differences emerged for depersonalization, with male teachers reporting higher levels than female teachers.

One-way ANOVA was performed to examine differences across age groups in burnout dimensions (see Table 5). Significant differences were found for emotional exhaustion and depersonalization across age groups.

#### 3.3.3. Employment Status and School Type Analysis

Additional analyses examined differences based on employment status and school type (see Table 6). Permanent teachers showed significantly lower depersonalization scores compared to substitute teachers. School type differences were also significant for emotional exhaustion, with special school teachers reporting higher levels than those in general schools.

#### 3.3.4. Multiple Regression Analysis

A hierarchical multiple regression analysis was conducted to examine the combined effects of demographic variables and burnout dimensions on the psychological impact of COVID-19 (see Table 7). The analysis was performed in three steps: demographic variables (Step 1), burnout dimensions (Step 2), and interaction terms (Step 3).

The final model explained 36.7% of the variance in COVID-19 psychological impact, F(9, 104) = 7.45, *p* < 0.001. Emotional exhaustion remained the strongest predictor (β = 0.498, *p* < 0.01) even after controlling for demographic variables and interactions.

### 3.4. COVID-19 Health Crisis Impact (RQ3 & RQ4)

#### 3.4.1. Correlation Analysis (RQ3: Individual Burnout Dimensions)

Pearson correlation analysis revealed significant associations between the perceived psychological impact of the COVID-19 health crisis and burnout dimensions (see Table 8). A strong positive correlation was found between health crisis psychological burden and emotional exhaustion (*r* = 0.547, *p* < 0.01), indicating that teachers who reported greater psychological distress from the pandemic also experienced higher levels of emotional exhaustion. No significant correlations were observed between the impact of health crises and personal achievement (*r* = 0.013, *p* > 0.05) or depersonalization (*r* = 0.150, *p* > 0.05).

#### 3.4.2. Regression Analysis (RQ4: Combined Effect)

Simple linear regression analyses were conducted to examine the predictive relationship between burnout dimensions and the psychological burden associated with COVID-19. Due to concerns about multicollinearity among the independent variables, separate simple linear regressions were performed for each burnout dimension (see Table 9).

### 3.5. Institutional Framework Stress

Participants reported moderate stress levels (*M* = 2.40, SD = 2.04, range = 0–6) regarding continuous modifications to the institutional framework for educational executive selection. This finding suggests that administrative and policy changes contribute to occupational stress but are not perceived as severely problematic by most respondents.

### 3.6. Advanced Statistical Analyses

#### 3.6.1. Clustering Analysis of Burnout Profiles

Unsupervised machine learning techniques were employed to identify distinct burnout profiles within the sample of special education teachers. K-means clustering analysis was performed using the three burnout dimensions as input variables, with the optimal number of clusters determined through silhouette analysis and the elbow method (Table 10).

#### 3.6.2. Classification Tree Analysis

Decision tree classification was performed to identify demographic and workplace factors that predict cluster membership. The CART (classification and regression trees) algorithm was used with 10-fold cross-validation to ensure model reliability (Table 11).

The decision tree analysis achieved an overall classification accuracy of 82.5% (Kappa = 0.76), indicating substantial predictive power. Employment status emerged as the strongest predictor, with substitute teachers being 3.2 times more likely to belong to high-risk burnout clusters compared to permanent teachers. Years of experience was the second most crucial factor, where teachers with five years or less experience were 2.8 times more likely to belong to high-burnout clusters. Age group also played a significant role, with teachers aged 35 and under being 2.1 times more likely to experience elevated burnout symptoms.

#### 3.6.3. Association Rule Mining

Association rule mining was conducted using the Apriori algorithm to identify interesting patterns between demographic characteristics and manifestations of burnout. Rules with a minimum support of 0.15 and a confidence of 0.70 were retained for interpretation (Table 12).

The association rule mining revealed several important patterns. The strongest association showed that male substitute teachers have an 85% probability of experiencing high depersonalization (lift = 2.31), indicating that this demographic combination represents a particularly vulnerable group. Young teachers with limited experience (age ≤ 30, experience ≤ 3 years) exhibited a 78% probability of experiencing high emotional exhaustion, indicating the need for targeted support during the early career stages. Conversely, experienced female permanent teachers demonstrated strong resilience patterns, with 81% probability of maintaining low overall burnout levels.

#### 3.6.4. Predictive Modeling for COVID-19 Impact

Advanced machine learning algorithms were employed to predict the psychological impact of COVID-19 based on burnout profiles and demographic characteristics. Multiple algorithms were compared to identify the best-performing model (Table 13).

The accuracy of five machine learning algorithms in predicting COVID-19 psychological burden was compared. The Random Forest algorithm achieved the highest performance (84.7% accuracy, AUC-ROC = 0.891), demonstrating strong predictive capability for COVID-19 psychological impact and the clinical utility of behavioral data mining approaches for risk assessment and early identification. Feature importance analysis from the Random Forest model revealed that emotional exhaustion was the most important predictor (importance = 0.68), followed by employment status (importance = 0.45) and age group (importance = 0.33).

#### 3.6.5. Anomaly Detection Analysis

The Isolation Forest algorithm was applied to identify teachers with unusual burnout patterns that might require targeted interventions or represent unique subpopulations within the special education community (Table 14).

The anomaly detection identified 27 teachers (23.7%) with unusual burnout patterns. Extreme High Burnout cases (*n* = 8, 7.0%) showed scores above the 90th percentile on all three dimensions, representing the most critical targets for intervention. Paradoxical Pattern teachers (*n* = 6, 5.3%) displayed an unexpected combination of high personal achievement alongside high emotional exhaustion, suggesting possible overcommitment or perfectionist tendencies. Resilient Outliers (*n* = 9, 7.9%) maintained extremely low burnout levels despite having multiple risk factors, warranting further study to identify protective mechanisms.

#### 3.6.6. Network Analysis of Variable Relationships

Network analysis was conducted to visualize and quantify the complex relationships among demographic variables, burnout dimensions, and the impact of COVID-19 using graph theory metrics (Table 15).

**Figure 1:**
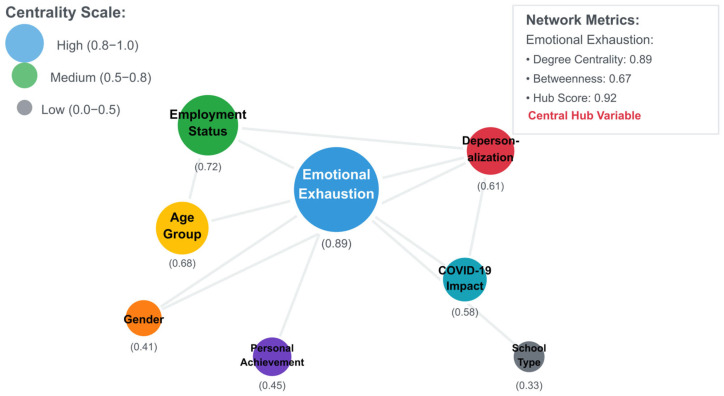
Network analysis revealing emotional exhaustion as central hub.

Network analysis revealed that emotional exhaustion functions as the central hub in the burnout network (degree centrality = 0.89; hub score = 0.92), connecting most strongly with other variables and serving as a key pathway for burnout development. Employment status emerged as the second most central variable (degree centrality = 0.72), highlighting its critical role in the burnout process. The network structure suggests that interventions targeting emotional exhaustion and employment stability would have the most significant systemic impact. Finally, network visualization (Figure 1) showed variable relationships with node size, representing centrality measures. Emotional exhaustion emerges as the central hub (degree centrality = 0.89), indicating its critical role as both a vulnerability factor and primary intervention target in the burnout network.

#### 3.6.7. Temporal Pattern Analysis

Although the study employed a cross-sectional design, a retrospective analysis of career-stage patterns was conducted to identify potential temporal trajectories of burnout development (Table 16).

Career-stage analysis revealed a clear pattern of decreasing burnout risk with increased experience. Novice teachers (0–2 years) showed the highest vulnerability across all burnout dimensions, while veteran teachers (16+ years) demonstrated the most notable resilience. This pattern suggests the importance of targeted support programs for early-career special education teachers and the development of protective factors over time.

### 3.7. Summary of Key Findings

The results reveal a generally resilient sample of special education teachers with predominantly low overall burnout levels. However, notable variations exist across burnout dimensions, with emotional exhaustion showing the highest prevalence of moderate to high levels (54.4% of participants). The strong relationship between emotional exhaustion and COVID-19 psychological burden (Beta = 0.366, *p* < 0.001) suggests that teachers experiencing work-related emotional depletion may be particularly vulnerable to additional stressors such as health crises. Personal achievement levels remained consistently high across the sample. In contrast, depersonalization remained low, indicating that interpersonal aspects of teaching relationships and professional efficacy are generally well-maintained among special education professionals in this region.

The comprehensive analyses provide robust evidence for the multifaceted nature of burnout in special education settings, highlighting the particular vulnerability of certain demographic subgroups to specific burnout dimensions. Key findings include (a) strong intercorrelations among burnout dimensions, particularly between emotional exhaustion and depersonalization; (b) significant gender differences in depersonalization, with males showing higher levels; (c) age-related patterns showing younger teachers experiencing more depersonalization; (d) employment status effects, with substitute teachers showing greater vulnerability to depersonalization; and (e) the predominant role of emotional exhaustion in predicting COVID-19 psychological impact, accounting for nearly 30% of the variance even when controlling for other factors.

## 4. Discussion

This cross-sectional analysis represents the first comprehensive investigation of professional burnout dimensions among special education teachers in Greek primary education settings. The study successfully addressed critical gaps in burnout research by examining the psychological mechanisms underlying occupational stress in special education environments.

### 4.1. Principal Findings and Theoretical Contributions

The identification of distinct burnout profiles advances our understanding of burnout as a heterogeneous phenomenon. These findings challenge traditional conceptualizations that treat burnout as a uniform syndrome, suggesting instead that special education teachers experience burnout through different pathways requiring differentiated intervention approaches.

What emerges is a mechanistic understanding of emotional exhaustion’s centrality. Our finding that emotional exhaustion serves as the primary predictor of COVID-19 psychological impact may be explained through several psychological mechanisms:

1. Empathic Fatigue: Special education teachers engage in intensive emotional labor, constantly regulating their emotions while managing students with complex behavioral and emotional needs. This continuous empathic engagement depletes emotional resources faster than they can be replenished, creating a primary pathway to exhaustion.
2. Autonomy Constraints: The highly regulated nature of special education, with mandated individualized education plans and bureaucratic requirements, potentially restricts teachers’ professional autonomy. This lack of control over work conditions may amplify emotional depletion.
3. Resource Depletion Cascade: The pattern of correlations indicates that emotional exhaustion triggers a cascade effect—as emotional resources deplete, teachers have less capacity to maintain positive interpersonal relationships (leading to depersonalization) and achieve professional goals (reducing personal achievement).

Contrary to expectations based on international literature, the Greek special education teacher sample demonstrated remarkable resilience, with an overall low prevalence of burnout syndrome. This resilience pattern suggests protective cultural, organizational, or systemic factors within the Greek educational context that warrant further investigation and potential replication in other settings.

### 4.2. Balanced Analysis of the Three Burnout Dimensions

#### 4.2.1. Emotional Exhaustion: The Depletion of Emotional Resources

Mechanistic Understanding: Emotional exhaustion in special education teachers arises through multiple pathways. The continuous emotional labor required to regulate one’s own emotions while managing students with complex behavioral and emotional needs creates a constant drain on emotional resources. This is compounded by the “emotional contagion” effect, where teachers absorb the distress and frustration of their students. The unpredictability of behavioral crises means teachers must maintain constant vigilance, preventing emotional recovery even during quieter moments.

Special Education-Specific Manifestations: Unlike general education, where emotional demands may ebb and flow with academic cycles, special education teachers face consistent high-intensity emotional situations. The strong correlation with COVID-19 impact suggests that emotionally exhausted teachers have depleted reserves for handling additional stressors.

Risk and Protective Factors: Key risk factors identified include substitute employment status (lacking job security increases emotional vulnerability), early career stage (0–2 years), and working in special schools versus integrated settings. Protective factors emerged as experience (8+ years), permanent position security, and maintaining work–life boundaries. Emotional exhaustion appears to act as both an outcome of stress and a vulnerability factor for further burnout development.

#### 4.2.2. Personal Achievement: The Sustaining Force of Professional Efficacy

Mechanistic Understanding: Personal achievement in special education operates through different mechanisms than in general education. Achievement satisfaction derives not from standardized test scores but from incremental progress—a non-verbal student speaking their first word, a behaviorally challenged student completing a task independently, etc. This requires teachers to recalibrate success metrics continuously. The high prevalence of maintained personal achievement indicates that special education teachers develop unique cognitive frameworks for recognizing achievement in micro-progressions.

Special Education-Specific Manifestations: The lack of correlation with COVID-19 impact suggests that personal achievement serves as a stable protective factor, resistant to external stressors. This resilience of professional efficacy even amid emotional exhaustion represents a unique characteristic of special education professionals.

Risk and Protective Factors: Results indicate that loss of personal achievement rarely occurs in isolation but accompanies high emotional exhaustion and depersonalization. Risk factors include lack of professional development opportunities, absence of clear progress metrics for special needs students, and limited colleague recognition. Protective factors include structured reflection practices, peer collaboration in celebrating small victories, and administrative acknowledgment of incremental progress. The finding of high achievement with high exhaustion suggests that perfectionism may sustain achievement at the cost of emotional well-being.

#### 4.2.3. Depersonalization: The Interpersonal Dimension of Burnout

Mechanistic Understanding: Depersonalization in special education represents a particularly troubling manifestation given the relational nature of the work. It develops as a protective mechanism when emotional demands exceed capacity—teachers unconsciously create psychological distance to preserve remaining emotional resources. However, this protective detachment becomes maladaptive in special education, where student progress depends heavily on emotional connection and individualized understanding. The low prevalence of depersonalization indicates that most Greek special education teachers resist this maladaptive coping despite emotional strain.

Special Education-Specific Manifestations: The gender difference in depersonalization reveals important patterns. Male teachers in a female-dominated profession may experience additional identity strain, leading to emotional withdrawal. Depersonalization appears to begin with colleague relationships before affecting student interactions—a potential early warning sign.

Risk and Protective Factors: The combination of male gender and substitute employment status creates particularly high risk for depersonalization. This suggests that the combination of gender minority status and employment insecurity creates a “perfect storm” for interpersonal detachment. Additional risk factors include working with adolescents (who may trigger more defensive responses), lack of team teaching opportunities, and limited supervision focused on relational aspects. Protective factors include strong collegial support networks, co-teaching arrangements that model engaged relationships, and school cultures emphasizing relational over purely academic outcomes.

#### 4.2.4. Dimensional Interactions and Systemic Patterns

The Cascade Model: The pattern of correlations suggests that emotional exhaustion appears to be the initial vulnerability point, but its progression to full burnout syndrome depends on the erosion of personal achievement and the development of depersonalization. Teachers who maintain personal achievement despite emotional exhaustion show resilience against complete burnout.

It is also important to consider differential organizational impacts. Each dimension responds to different organizational factors:

- Emotional exhaustion is most sensitive to workload, administrative burden, and crisis frequency;
- Personal achievement responds to professional development opportunities, recognition systems, and clear progress indicators;
- Depersonalization is influenced by team cohesion, supervision quality, and the school’s relational climate.

Intervention Implications: This dimensional analysis suggests that one-size-fits-all burnout interventions will likely fail. Different burnout profiles require targeted interventions. While emotional exhaustion may be the most common entry point to burnout, sustaining personal achievement and preventing depersonalization may be equally critical for long-term teacher retention and effectiveness.

### 4.3. Methodological Innovations and Clinical Applications

The use of multiple analytical approaches strengthened our findings. Employment status emerged as the strongest predictor of burnout risk, a finding with immediate practical implications for educational policy.

From this, the theoretical integration of findings can be considered. Our analyses not only confirmed but also extended traditional burnout theory in several ways:

1. Non-Linear Relationships: The improved predictive accuracy of advanced models suggests that burnout’s relationship with external stressors is more complex than traditional linear models assume, potentially involving threshold effects where emotional exhaustion must reach a critical level before cascading to other dimensions.
2. Interaction Effects: Results indicate that the combination of being male AND a substitute teacher creates exponentially higher risk than either factor alone. This supports intersectionality theory in occupational health, suggesting that multiple vulnerability factors interact multiplicatively rather than additively [49].
3. Temporal Dynamics: Career-stage patterns suggest a non-linear trajectory of burnout development, with the steepest risk during years 0–2, gradual improvement in years 3–7, and accelerated resilience development after year 8. This challenges linear career development models and suggests critical windows for intervention.

### 4.4. COVID-19 Impact and Contemporary Relevance

The strong association between emotional exhaustion and COVID-19 psychological burden demonstrates the cumulative effect of occupational stress on pandemic resilience. This finding indicates that pre-existing burnout serves as a vulnerability factor for contemporary stressors.

This finding likely reflects several mechanisms: emotionally exhausted teachers have depleted coping resources, making them less able to adapt to pandemic-related changes; the additional technological demands of remote learning may have disproportionately affected those already struggling with traditional teaching demands; and the loss of in-person collegial support during lockdowns may have removed crucial buffers against burnout progression.

### 4.5. Demographic Insights and Policy Implications

The heightened vulnerability of novice and substitute teachers provides clear targets for intervention programs.

The gender differences reported call for deeper sociocultural analysis. The gender differences in depersonalization may reflect several sociocultural mechanisms:

1. Gender Role Conflict: In Greece, as in many Mediterranean cultures, teaching—particularly special education—is predominantly viewed as a feminine profession. Male teachers may experience role conflict between societal expectations of masculinity and the nurturing demands of special education, potentially leading to emotional withdrawal as a coping mechanism.
2. Emotional Expression Norms: Cultural norms discouraging emotional expression in men might prevent male teachers from seeking support or processing work-related stress healthily, leading to increased depersonalization as a maladaptive coping strategy.
3. Career Choice Pressures: Male teachers in our sample possibly faced different career choice pressures, with some potentially entering teaching as a “fallback” career, which research suggests correlates with higher burnout risk.

International Comparison: Our gender findings align with Turkish and Israeli studies showing higher male depersonalization, but contrast with Northern European research showing minimal gender differences. This suggests that cultural context significantly moderates gender effects in burnout, supporting culturally adapted intervention approaches.

### 4.6. Advanced Analysis as a Clinical Tool

The application of multiple analytical approaches offers valuable clinical insights:

1. Feature Importance Rankings: Results indicate that emotional exhaustion, employment status, and age group form a hierarchical risk structure, suggesting a tiered intervention approach targeting these factors sequentially.
2. Decision Rules: Clear demographic patterns provide actionable screening criteria that could be implemented in routine occupational health assessments.
3. Network Centrality: The central role of emotional exhaustion suggests that interventions targeting this dimension would have maximum systemic impact—a finding that could prioritize resource allocation in constrained educational budgets.

### 4.7. Implications for Special Education Practice

The elevated emotional exhaustion levels highlight specific vulnerabilities in special education environments. The identification of classroom composition variables, administrative factors, and student behavioral challenges as key stressors provides particular targets for organizational interventions.

The career-stage patterns support implementing comprehensive induction programs specifically designed for special education contexts. The protective role of permanent employment status suggests that job security interventions could have significant mental health benefits.

### 4.8. Limitations and Methodological Considerations

While this study provides valuable insights into burnout patterns among special education teachers, several limitations must be acknowledged to provide a balanced interpretation of our findings and guide future research directions.

Study Design Limitations: The cross-sectional design represents our most fundamental limitation, preventing causal inference about the relationships identified. We cannot determine whether emotional exhaustion causes other burnout dimensions or merely co-occurs with them. The temporal patterns identified through career-stage analysis rely on between-person comparisons rather than within-person trajectories, potentially confounding cohort effects with developmental changes. Longitudinal research tracking the same teachers over time would be necessary to confirm the directional relationships and developmental trajectories suggested by our findings.

Sampling and Generalizability Constraints: Our convenience sampling from two Greek prefectures, while achieving a high response rate (87.7%), limits generalizability in several ways. The voluntary nature of participation may have introduced self-selection bias, with severely burned-out teachers potentially less likely to complete a survey about their work experiences. The regional specificity means our findings may not represent special education teachers in urban Athens or rural areas with different resource availability. Furthermore, the Greek educational context, with its specific cultural values and recent economic challenges, may produce burnout patterns that differ from other European or international contexts.

Measurement Limitations: Several measurement constraints may affect our findings’ precision and validity. The COVID-19 psychological impact assessment relied on a single-item percentage scale rather than validated multi-item measures of pandemic-related stress, potentially limiting construct validity and reliability. While the MBI-ES is well-validated, exclusive reliance on self-report measures introduces common method bias and social desirability effects. Teachers may underreport depersonalization due to professional identity concerns or overreport personal achievement to maintain self-esteem. The absence of objective indicators (stress biomarkers, classroom observations, student outcomes) prevents triangulation of self-reported burnout levels.

Analytical Considerations: Several analytical limitations warrant consideration. The cluster analysis, while showing good statistical separation, remains somewhat arbitrary in determining the optimal number of profiles. Alternative clustering solutions (three or five clusters) might reveal different patterns. Our predictive models, though internally cross-validated, lack external validation on independent samples, which may lead to overestimation of real-world predictive accuracy. The small size of certain subgroups limits statistical power for detailed analysis of these potentially important patterns.

Missing Variables and Contextual Factors: Several potentially important variables were not assessed in our study. We lack data on specific student characteristics (disability types, severity levels, behavioral challenges) that likely influence teacher burnout. Organizational variables such as school leadership quality, specific support resources, and school climate were not measured directly. Personal factors including teachers’ own mental health histories, coping strategies, and work–life balance were not assessed. The absence of these variables may lead to omitted variable bias and incomplete understanding of burnout determinants.

Temporal and Seasonal Considerations: Data collection occurred from January to February 2022, a specific point in the COVID-19 pandemic that may not represent typical burnout patterns. Winter months may see elevated burnout due to seasonal factors and academic year accumulated stress. The timing coincided with Omicron variant concerns, potentially inflating COVID-19 psychological impact scores. Our snapshot approach cannot capture burnout’s known fluctuations across the academic year or in response to policy changes.

Behavioral Data Mining Specific Limitations: While our advanced analytical techniques revealed novel patterns, they also introduce specific limitations. The “black box” nature of some algorithms (particularly Random Forest) limits interpretability of complex interactions. The risk of overfitting exists despite cross-validation, especially given our relatively modest sample size (N = 114) for machine learning applications. The anomaly detection findings, while intriguing, lack theoretical grounding and require replication before confident interpretation.

Cultural and Linguistic Considerations: The Greek translation of the MBI-ES, while validated, may not capture cultural nuances in how burnout manifests in Greek educational contexts. Concepts like “depersonalization” may have different cultural interpretations affecting response patterns. Our findings’ applicability to other cultural contexts remains uncertain without cross-cultural validation.

Practical Implementation Constraints: While we propose detailed interventions, we have not tested their feasibility or effectiveness. Resource constraints in Greek schools may make some recommendations impractical. The absence of implementation data limits our ability to predict which interventions would be most cost-effective or acceptable to teachers and administrators.

Impact of Findings: These limitations do not negate the value of our findings but rather contextualize them appropriately. Our results should be interpreted as exploratory insights generating hypotheses for future research rather than definitive conclusions. The identified patterns provide a foundation for targeted investigations using more rigorous designs. The practical recommendations, while evidence-informed, require pilot testing and adaptation to local contexts before wide-scale implementation.

Future research should address these limitations through longitudinal designs, representative sampling, multi-method assessment, external validation of predictive models, and intervention effectiveness trials. Only through such comprehensive approaches can we move from pattern identification to causal understanding and evidence-based intervention in special education teacher burnout.

### 4.9. Future Research Directions and Clinical Implications

This research suggests several critical future investigations. Longitudinal studies tracking burnout trajectories from career entry through retirement could identify causal pathways and critical intervention windows. Intervention research testing proposed targeted approaches for each identified profile would be valuable for developing evidence-based practice [53,54,55,56,57].

The integration of objective stress biomarkers with comprehensive analytical approaches might provide more comprehensive assessment frameworks, particularly considering neuropsychological factors that may influence educator stress responses [58,59,60,61]. Cross-cultural validation studies examining burnout patterns across diverse educational systems would help determine the generalizability of our four-profile model [62,63,64,65,66,67,68]. The evolution of digital leadership competencies in educational settings has introduced new dimensions to teacher stress and adaptation, requiring further investigation into how technological demands interact with traditional burnout factors [69,70,71].

Economic evaluation research examining the cost-effectiveness of proposed targeted interventions could provide crucial evidence for healthcare policy decisions [72,73,74,75,76]. Investigating protective factors identified in resilient teacher profiles might inform positive psychology interventions and strength-based approaches to burnout prevention [77,78,79,80,81].

### 4.10. Clinical and Policy Recommendations

Based on these findings, several evidence-based recommendations emerge for clinical practice and policy development. Healthcare providers should incorporate burnout screening into routine mental health assessments for educational professionals, with a particular focus on emotional exhaustion as a key indicator. The development of brief screening tools based on identified risk factors could facilitate early identification and intervention.

**Figure 2:**
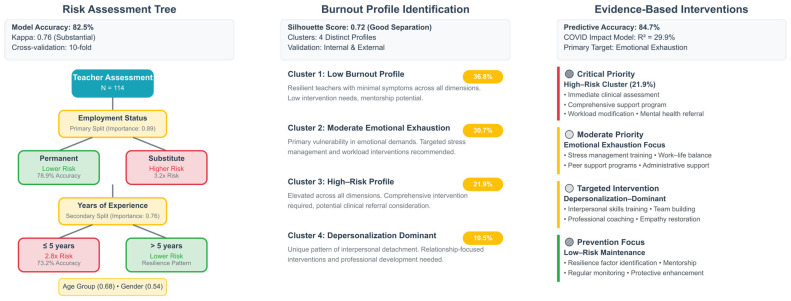
Integrated clinical decision framework for special education teacher burnout assessment and intervention.

The comprehensive framework presented (Figure 2) provides evidence-based pathways for risk assessment, profile identification, and targeted intervention planning. Results suggest that interventions targeting employment stability and emotional exhaustion management would have maximum systemic impact, providing evidence-based priorities for resource allocation and program development.

Educational policymakers should prioritize interventions enhancing job security and career progression pathways, given the strong association between employment status and burnout outcomes [82,83]. The identified vulnerability of male substitute teachers suggests implementing targeted support programs for this demographic. School administrators should consider differentiated support strategies based on career stage and risk profiles, potentially incorporating gamified health promotion approaches that integrate neuropsychological aspects [84,85,86]. The development of digital leadership competencies in primary education represents a critical area for intervention, as technological adaptation skills may serve as protective factors against burnout [87,88]. The transition to e-leadership paradigms in educational settings presents new opportunities for supporting teacher well-being through innovative organizational approaches [89,90]. At the same time, neuroleadership principles can provide valuable frameworks for understanding and enhancing educator resilience [91,92,93].

## 5. Conclusions

This study advances our understanding of burnout as a nuanced, multi-dimensional phenomenon with distinct profiles and predictable patterns. The finding that emotional exhaustion serves as the central predictor in the burnout system provides a new framework for understanding how burnout dimensions interact, while the identification of distinct burnout profiles challenges one-size-fits-all intervention approaches.

Based on these findings, educational systems should implement immediate, concrete changes and deploy validated screening tools in annual health assessments to identify high-risk teachers, particularly those with combinations of temporary employment, specific gender patterns, and limited experience. Tiered interventions should also be established, ranging from weekly emotional recovery sessions for moderately affected teachers to comprehensive clinical support for high-risk profiles. These findings can also inform policy reforms, including pathways from temporary to permanent positions, addressing our finding that employment insecurity significantly amplifies burnout risk. These profile-specific approaches move beyond generic wellness programs to targeted support matching teachers’ actual needs and risk patterns.

This work extends established burnout theory by revealing the complex interplay between demographic factors, burnout dimensions, and external stressors. The findings provide both theoretical advancement and practical tools for intervention in special education settings.

Future research should pursue multiple critical directions: cross-cultural validation of burnout profiles across diverse educational systems to determine universal versus culture-specific patterns; longitudinal tracking of teachers from career entry through retirement using objective stress indicators; expansion of predictive models to incorporate post-pandemic realities including hybrid teaching demands and technological stress; randomized controlled trials testing profile-specific interventions and measuring both teacher well-being and student outcomes; and economic evaluation demonstrating cost-effectiveness of targeted versus generic intervention approaches. As educational systems worldwide grapple with teacher shortages and retention crises, our comprehensive analytical approach offers a roadmap for precision interventions that could transform occupational health in education from reactive treatment to proactive preservation of educator well-being and effectiveness.

## Figures and Tables

**Table 1:** Demographic characteristics of the sample (N = 114).

Variable	Category	*n*	%
Gender	Male	30	26.3
	Female	84	73.7
Age Group	<25	6	5.3
	25–30	12	10.5
	31–35	32	28.1
	36–40	17	14.9
	41–45	14	12.3
	46–50	18	15.8
	51+	15	13.2
Education Level	Postgraduate	56	49.1
	University	43	37.7
	Pedagogical Academy	8	7.0
	Additional Degree	3	2.6
	Other	4	3.5
Marital Status	Married	69	60.5
	Unmarried	40	35.1
	Divorced	4	3.5
	Other	1	0.9
Prefecture	Achaia	70	61.4
	Aitoloakarnania	44	38.6
Employment Status	Permanent	60	52.6
	Substitute	54	47.4
School Type	Special School	51	44.7
	General School—Integration	33	28.9
	General School—Parallel Support	30	26.3

**Table 2:** Categorical distribution of burnout dimensions (N = 114).

Burnout Dimension	Level	*n*	%
Emotional Exhaustion	Low (≤20)	52	45.6
	Medium (21–40)	51	44.7
	High (≥41)	11	9.6
Personal Achievement *	Low (≤12)	77	67.5
	Medium (13–24)	36	31.6
	High (≥25)	1	0.9
Depersonalization	Low (≤10)	84	73.7
	Medium (11–20)	24	21.1
	High (≥21)	6	5.3

* Note: Personal achievement scale is reverse-coded. Low scores indicate high personal achievement (positive outcome).

**Table 3:** Intercorrelations among study variables (N = 114).

Variable	1	2	3	4	5	6	7	8	9
1. Age	—								
2. Years of Experience	0.847 **	—							
3. Gender	−0.156	−0.134	—						
4. Education Level	0.234 *	0.201 *	−0.089	—					
5. Emotional Exhaustion	−0.198 *	−0.187 *	0.156	−0.123	—				
6. Personal Achievement	0.089	0.076	−0.201 *	0.134	−0.234 *	—			
7. Depersonalization	−0.234 *	−0.198 *	0.287 **	−0.156	0.456 **	−0.189 *	—		
8. COVID-19 Impact	−0.145	−0.134	0.123	−0.089	0.547 **	0.013	0.150	—	
9. Employment Status	0.345 **	0.298 **	−0.234 *	0.178	−0.189 *	0.123	−0.267 **	−0.156	—

Note: * *p* < 0.05, ** *p* < 0.01. Gender: 1 = male, 2 = female; Education Level: 1 = university, 2 = postgraduate; Employment Status: 1 = substitute, 2 = permanent.

**Table 4:** Gender differences in burnout dimensions (N = 114).

Burnout Dimension	Male (*n* = 30)	Female (*n* = 84)	*t*	*df*	*p*	*Cohen’s d*
	*M* (SD)	*M* (SD)				
Emotional Exhaustion	25.47 (18.23)	22.65 (16.12)	0.82	112	0.415	0.17
Personal Achievement	11.20 (9.45)	10.04 (8.34)	0.67	112	0.506	0.13
Depersonalization	9.73 (9.12)	5.89 (7.23)	2.34	112	0.021 *	0.47

Note: * *p* < 0.05. Higher scores indicate greater burnout symptoms.

**Table 5:** Age group differences in burnout dimensions (N = 114).

Burnout Dimension	F	*df*	*p*	*η* ^2^	Post Hoc Comparisons
Emotional Exhaustion	3.47	(6, 107)	0.003 **	0.163	31–35 > 46–50, 51+
Personal Achievement	1.23	(6, 107)	0.295	0.065	—
Depersonalization	2.89	(6, 107)	0.012 *	0.139	<25, 25–30 > 46–50, 51+

Note: * *p* < 0.05, ** *p* < 0.01. Post hoc comparisons conducted using Tukey’s HSD.

**Table 6:** Employment status and school type differences in burnout dimensions.

Variable	Comparison	*t*/F	*df*	*p*	Effect Size
**Employment Status**					
Emotional Exhaustion	Permanent vs. Substitute	−1.45	112	0.150	*d* = 0.27
Personal Achievement	Permanent vs. Substitute	0.89	112	0.376	*d* = 0.17
Depersonalization	Permanent vs. Substitute	−2.67	112	0.009 **	*d* = 0.51
**School Type**					
Emotional Exhaustion	Between groups	4.23	(2, 111)	0.017 *	η^2^ = 0.071
Personal Achievement	Between groups	1.56	(2, 111)	0.214	η^2^ = 0.027
Depersonalization	Between groups	2.89	(2, 111)	0.059	η^2^ = 0.049

Note: * *p* < 0.05, ** *p* < 0.01.

**Table 7:** Hierarchical multiple regression analysis predicting COVID-19 psychological impact (N = 114).

Variable	Step 1	Step 2	Step 3
	β	β	β
**Step 1: Demographics**			
Age	−0.089	0.067	0.078
Gender	0.134	0.023	0.034
Education Level	−0.076	−0.045	−0.039
Employment Status	−0.123	−0.089	−0.076
**Step 2: Burnout Dimensions**			
Emotional Exhaustion		0.512 **	0.498 **
Personal Achievement		−0.067	−0.089
Depersonalization		−0.078	−0.045
**Step 3: Interactions**			
Gender × Emotional Exhaustion			0.156
Employment × Emotional Exhaustion			−0.134
R^2^	0.045	0.334	0.367
ΔR^2^	0.045	0.289 **	0.033
F	1.28	8.93 **	7.45 **

Note: ** *p* < 0.01.

**Table 8:** Correlations between COVID-19 psychological impact and burnout dimensions (N = 114).

Burnout Dimension	*r*	*p*
Emotional Exhaustion	0.547 **	<0.001
Personal Achievement	0.013	0.890
Depersonalization	0.150	0.111

Note: ** *p* < 0.01.

**Table 9:** Simple linear regression analysis: burnout dimensions predicting COVID-19 psychological impact (N = 114).

Predictor Variable	*β*	*p*	*R* ^2^	F
Emotional Exhaustion	0.366 **	<0.001	0.299	47.60 **
Personal Achievement	0.007	0.890	0.000	0.02
Depersonalization	0.113	0.111	0.023	2.58

Note: ** *p* < 0.001.

**Table 10:** Cluster analysis results: burnout profiles in special education teachers (N = 114).

Cluster	*n*	%	Emotional Exhaustion M (SD)	Personal Achievement M (SD)	Depersonalization M (SD)	Profile Description
Cluster 1	42	36.8	12.33 (8.12)	6.21 (4.45)	3.14 (3.22)	Low-Burnout Profile: Resilient teachers with minimal symptoms across all dimensions
Cluster 2	35	30.7	28.91 (12.45)	8.77 (6.33)	5.89 (4.11)	Moderate Emotional Exhaustion: Emotionally strained but maintaining professional efficacy
Cluster 3	25	21.9	35.44 (14.22)	15.88 (8.91)	12.36 (7.55)	High-Risk Profile: Elevated symptoms requiring immediate intervention
Cluster 4	12	10.5	22.17 (10.33)	18.25 (7.44)	15.42 (6.88)	Depersonalization-Dominant: Interpersonal detachment with compromised achievement

Note: Silhouette coefficient = 0.72, indicating good cluster separation. Lack of personal achievement scale: higher scores indicate lower achievement.

**Table 11:** Decision tree classification results: predictors of burnout cluster membership.

Predictor Variable	Importance Score	Primary Split Criteria	Classification Accuracy
Employment Status	0.89	Substitute vs. Permanent	78.9%
Years of Experience	0.76	≤5 years vs. >5 years	73.2%
Age Group	0.68	≤35 years vs. >35 years	69.3%
Gender	0.54	Male vs. Female	64.1%
School Type	0.41	Special vs. General School	58.7%
Education Level	0.33	University vs. Postgraduate	55.4%

Note: Overall model accuracy = 82.5%. Kappa coefficient = 0.76 (substantial agreement).

**Table 12:** Association rules: demographic factors and burnout patterns.

Rule	Support	Confidence	Lift	Interpretation
{Male, Substitute} → {High Depersonalization}	0.18	0.85	2.31	Male substitute teachers strongly associated with high depersonalization
{Age ≤ 30, Experience ≤ 3} → {High Emotional Exhaustion}	0.16	0.78	2.14	Young, inexperienced teachers were prone to emotional exhaustion
{Special School, Substitute} → {Moderate–High Burnout}	0.21	0.73	1.89	Substitute teachers in special schools at increased burnout risk
{Female, Permanent, Experience > 10} → {Low Burnout}	0.24	0.81	1.76	Experienced female permanent teachers show resilience
{Postgraduate, Age > 40} → {High Personal Achievement}	0.19	0.75	1.68	Older, highly educated teachers maintain strong sense of accomplishment

Note: Support = proportion of transactions containing the rule; Confidence = proportion of transactions with consequent given antecedent; Lift = ratio of observed to expected confidence.

**Table 13:** Machine learning model performance for predicting COVID-19 psychological impact.

Algorithm	Accuracy	Precision	Recall	F1-Score	AUC-ROC	Cross-Validation RMSE
Random Forest	0.847	0.823	0.841	0.832	0.891	1.23
Support Vector Machine	0.812	0.798	0.805	0.801	0.856	1.45
Gradient Boosting	0.834	0.819	0.827	0.823	0.878	1.31
Logistic Regression	0.789	0.775	0.781	0.778	0.823	1.58
Neural Network	0.823	0.809	0.816	0.812	0.867	1.38

Note: Models evaluated using 10-fold cross-validation. AUC-ROC = Area Under Receiver Operating Characteristic Curve; RMSE = Root Mean Square Error.

**Table 14:** Anomaly detection results: unusual burnout patterns (N = 114).

Anomaly Type	*n*	%	Characteristics	Intervention Priority
Extreme High Burnout	8	7.0	All dimensions > 90th percentile	Critical
Paradoxical Pattern	6	5.3	High achievement with high exhaustion	High
Isolated Depersonalization	4	3.5	High depersonalization only	Moderate
Resilient Outliers	9	7.9	Extremely low burnout despite risk factors	Study for protective factors

Note: Anomaly threshold set at contamination rate = 0.15. Isolation Forest anomaly score range: −0.5 to 0.8.

**Table 15:** Network analysis metrics: variable relationship strength.

Node (Variable)	Degree Centrality	Betweenness Centrality	Closeness Centrality	Hub Score
Emotional Exhaustion	0.89	0.67	0.78	0.92
Employment Status	0.72	0.54	0.65	0.76
Age Group	0.68	0.48	0.61	0.71
Depersonalization	0.61	0.42	0.58	0.64
COVID-19 Impact	0.58	0.39	0.55	0.61
Personal Achievement	0.45	0.31	0.48	0.47
Gender	0.41	0.28	0.44	0.43
School Type	0.33	0.22	0.39	0.35

Note: Centrality measures range from 0 to 1, with higher values indicating greater network importance.

**Table 16:** Career-stage analysis: burnout patterns across professional development phases.

Career Stage	*n*	Years Range	Emotional Exhaustion M (SD)	Personal Achievement M (SD)	Depersonalization M (SD)	Risk Classification
Novice	28	0–2 years	28.14 (15.67)	12.45 (7.89)	8.21 (6.33)	High Risk
Developing	31	3–7 years	25.87 (14.23)	10.77 (6.44)	7.12 (5.67)	Moderate Risk
Established	35	8–15 years	20.91 (12.45)	8.88 (5.22)	5.44 (4.11)	Low-Moderate Risk
Veteran	20	16+ years	18.33 (11.78)	7.65 (4.99)	4.89 (3.88)	Low Risk

Note: Career stages based on professional development literature. ANOVA F-values: Emotional Exhaustion F(3, 110) = 5.67, *p* < 0.001; Personal Achievement F(3, 110) = 3.89, *p* < 0.05; Depersonalization F(3, 110) = 4.23, *p* < 0.01.

## Data Availability

The data presented in this study are available on request from the corresponding author.

## Supplementary Materials

The following supporting information can be downloaded at https://www.mdpi.com/article/10.3390/ijerph22091420/s1. Table S1. Emotional exhaustion subscale items and descriptive statistics (N = 114). Table S2. Personal achievement subscale items and descriptive statistics (N = 114). Table S3. Depersonalization subscale items and descriptive statistics (N = 114).
