# Supplementary material for: Psychological Dimensions of Professional Burnout in Special Education: A Cross-Sectional Behavioral Data Analysis of Emotional Exhaustion, Personal Achievement, and Depersonalization
Source: Int J Environ Res Public Health. 2025 Sep 11;22(9):1420. doi: 10.3390/ijerph22091420 (PMC12469340; doi:10.3390/ijerph22091420)
Supplement: Supplementary file 1 [file ijerph-22-01420-s001.zip › ijerph-3741902-supplementary.pdf]

Supplementary Materials – Tables S1, S2, and S3

3.2.1. Emotional Exhaustion

Analysis of the emotional exhaustion subscale revealed a mean total score of 23.31 (*SD* = 16.74, range = 0-60), indicating moderate levels of emotional exhaustion among participants. As detailed in Table S1, the highest-scoring item was "I feel like I'm working too hard at school" (*M* = 3.40, *SD* = 1.77), while the lowest was "I feel full of energy" (*M* = 1.74, *SD* = 1.34). Internal consistency for this subscale was excellent (Cronbach's α = .865).

Table S1. Emotional exhaustion subscale items and descriptive statistics (N = 114).

Item	*M*	*SD*	*Range*
I feel exhausted when I wake up in the morning and have to face another day of work	2.69	1.64	0-6
It's very tiring for me to work with people all day	2.24	1.76	0-6
I feel exhausted from my work	2.55	1.68	0-6
I feel like I'm working too hard at school	3.40	1.77	0-6
Working closely with teachers creates a lot of tension/stress for me	2.05	1.67	0-6
I feel like I'm at the limits of my endurance	2.15	1.57	0-6
I feel "empty" at the end of a school day	2.11	1.78	0-6
I feel full of energy*	1.74	1.34	0-6
I feel refreshed when I work with teachers*	1.98	1.50	0-6
The constant modification of the institutional framework creates tension/stress	2.40	2.04	0-6
Total Subscale Score	23.31	16.74	0-60

Note: Items marked with * are reverse-scored. Cronbach's α = .865.

3.2.2. Personal Achievement

The personal achievement subscale, reverse-coded to measure lack of personal achievement for consistency with other burnout dimensions, yielded a mean total score of 10.33 (SD = 8.64, range = 0-34). Lower scores on this reversed scale indicate higher personal achievement (a positive outcome). As shown in Table S2, the highest-scoring item was "I deal with teachers' problems very effectively" (M = 2.18, SD = 1.48), while the lowest was "I can create a comfortable atmosphere with teachers" (M = 1.41, SD = 1.35). The subscale demonstrated good internal consistency (Cronbach's α = .794).

Table S2. Personal achievement subscale items and descriptive statistics (N = 114).

Item	*M*	*SD*	*Range*
I deal with teachers' problems very effectively	2.18	1.48	0-6
I feel that my work has a positive impact on the lives of others	1.61	1.47	0-6
I can create a comfortable atmosphere with teachers	1.41	1.35	0-6
I have achieved many remarkable things in this job	1.85	1.30	0-6
I deal calmly with the problems that arise from my work	1.68	1.43	0-6
I can easily understand how the teachers in my school feel	1.60	1.62	0-6
**Total Subscale Score (Lack of Achievement)**	**10.33**	**8.64**	**0-34**

Note: Scale is reverse-coded. Lower scores indicate higher personal achievement (positive outcome). Higher scores indicate greater lack of personal achievement (burnout symptom). Cronbach's α = .794.

3.2.3. Depersonalization

The depersonalization subscale produced a mean total score of 6.79 (SD = 7.84, range = 0-29), indicating low levels of depersonalization across the sample. Table S3 shows that the highest-scoring item was "I don't really care what happens to some of the teachers" (M = 1.60, SD = 1.73), while the lowest

was "I feel that teachers blame me for some of their problems" (M = 1.12, SD = 1.41). Internal consistency was satisfactory (Cronbach's α = .794).

Table S3. Depersonalization subscale items and descriptive statistics (N = 114).

Item	*M*	*SD*	*Range*
I feel like I treat some teachers impersonally as if they were objects	1.24	1.61	0-6
I've become tougher on people since I started this job	1.32	1.44	0-6
I'm worried that this job will make me tougher	1.51	1.65	0-6
I don't really care what happens to some of the teachers	1.60	1.73	0-6
I feel that teachers blame me for some of their problems	1.12	1.41	0-6
**Total Subscale Score**	**6.79**	**7.84**	**0-29**

Note: Cronbach's α = .794.
